# Assessing the need to implement mass drug administration against *Wuchereria bancrofti* infection using both human serology and xenomonitoring in the urban conurbation of Monrovia, Liberia

**DOI:** 10.1371/journal.pntd.0013446

**Published:** 2025-09-18

**Authors:** Benjamin G. Koudou, Rogers Nditanchou, Firmain N. Yokoly, Abakar Gankpala, Karsor K. Kollie, David Molyneux, Philip Downs, Ruth Dixon

**Affiliations:** 1 Centre Suisse de Recherches Scientifiques en Côte d’Ivoire, Abidjan, Côte d’Ivoire; 2 UFR Sciences de la Nature, Université Nangui Abrogoua, Abidjan, Côte d’Ivoire; 3 Sightsavers, Haywards Heath, United Kingdom; 4 Sightsavers, Monrovia, Liberia; 5 Health Services Department, Neglected Tropical Diseases Program, Ministry of Health, Liberia; 6 Department of Tropical Disease Biology, Liverpool School of Tropical Medicine, Liverpool, United Kingdom; RTI International, UNITED STATES OF AMERICA

## Abstract

**Background:**

Lymphatic filariasis (LF) is a parasitic disease-causing severe pain, disfiguring, and disabling clinical conditions such as lymphoedema and hydrocoele that are associated with morbidity and stigma. The disease has been targeted for global elimination with the annual mass drug administration (MDA) strategy. We have evaluated the need to implement mass drug administration against *W. bancrofti* infection in urban zones of Monrovia using both serology and molecular Xenomonitoring (XM).

**Methodology:**

Confirmatory mapping protocols recommended by WHO were carried out in the four health districts of Monrovia. Schools were selected using probability proportionate to size (PPS) and eligible children aged 9–14 years were tested for circulating filarial antigen (CFA) using an Alere Filariasis Test Strip (FTS). Health Districts were assessed as requiring MDA if they exceeded the critical cut off of 3 positive tests corresponding to CFA ≥ 2%. Two health districts were selected for entomological investigations based on pre-disposing risk factors for mosquitoes. Mosquito collection was carried out using exit traps (ETs) and gravid trap (GTs) for 6 months. Mosquitos were tested for *W. bancrofti* DNA using qPCR.

**Principal findings:**

Ninety-six children in the sample had a positive serology test result, with a mean CFA prevalence of 5.3% (95% CI: 4.4 - 6.5%). All four health districts exceeded the critical cut off of 3 cases and in Somalia Drive there were 59 positive tests. In Central Monrovia which had 4 cases, 2 of them are likely locally imported from Somalia Drive where the children reported living. A total of 19,355 potential vector mosquitoes were collected, of which 84.4% (16,335) were *Culex* and 16.6% (3,020) *An. gambiae*. All mosquitoes were analyzed, and none were found to be infected with *W. bancrofti*.

**Conclusion:**

MDA is required in three health districts of Monrovia. Confirmatory mapping protocols require adaptation for urban settings. The sampling strategy for the XM was unable to identify transmission in this case and requires further research to optimise it for informing MDA implementation decisions.

## Author summary

Lymphatic filariasis (LF) remains a public health concern in endemic countries, with elimination targeted by 2030 through mass drug administration (MDA) and morbidity management. In low-prevalence and urban settings, decision-making is complicated by population density, operational challenges, and potential importation of cases. This study assessed the need for MDA in four health districts of Monrovia, Liberia using WHO’s confirmatory mapping protocol based on circulating filarial antigen (CFA) in children aged 9–14, alongside XM. All districts exceeded the CFA threshold for MDA (≥2%), although some positive cases were likely imported. Despite collecting over 19,000 potential vector mosquitoes, no *W. bancrofti* DNA was detected by XM, indicating discordant results between the two methods. This discrepancy likely stems from insufficient geographic alignment between mosquito and CFA sampling sites. The findings suggest that, while XM has potential as a complementary tool, optimized and spatially representative sampling strategies are essential, especially in complex urban environments. The study highlights the need for adapted confirmatory mapping protocols tailored to urban contexts to support accurate and cost-effective MDA decision-making.

## Background

Lymphatic filariasis (LF) is one of the most debilitating, disfiguring and stigmatizing diseases of the tropical and subtropical regions of the world. LF is endemic in more than 80 countries. It is estimated that 120 million people are infected and one-third of them suffer from chronic manifestations of the disease [1]. Recognizing the importance of the disease, the World Health Organization (WHO) launched the Global Program to Eliminate Lymphatic Filariasis (GPELF) in 2000, with the goal of 90% reduction in the population that requires interventions for the diseases (NTDs) by 2030 [2], through morbidity management and MDA involving combinations of albendazole, ivermectin, and/or diethylcarbamazine depending on setting and co-endemicities [3]. In general, WHO recommends starting MDA implementation in districts (or other implementation units) where antigenemia or microfilaremia (MF) prevalence is ≥ 1% (based on a convenience sample of adults in purposively selected locations) [4], and it is anticipated that 5–6 rounds of MDA should be sufficient to interrupt LF transmission [5–7]. After sufficient rounds of effective MDA transmission assessment surveys (TAS) are used to determine if infections have been reduced below thresholds that can sustain transmission indicating MDA can stop [8]. Antigenemia prevalence (also known as circulating filarial antigen (CFA) rate) can be determined using Filariasis Test Strips (FTS) or Immunochromatographic Test Cards (ICT) which have been demonstrated to be sufficiently sensitive and specific [9].

The focal nature of LF can mean that in low prevalence settings and especially in large implementation units the need for MDA can be indicated even though substantial areas may not have transmission [9]. Large urban conurbations within implementation units, present similar challenges; they are frequently included in the treatment decisions of larger predominantly rural districts, often without substantial evidence of localized transmission or disease prevalence. Recent operational research studies, mapping, and transmission assessment surveys conducted in large cities of West Africa [10–12] and Haiti [13] have shown that in comparison to surrounding peri-urban and rural areas urban areas had much lower CFA or MF and often did not reach the treatment threshold. Furthermore, as urban areas are often the focus of rural-urban migration, particularly in countries where there has been civil unrest or conflict [14], traditional markers may not reflect local transmission but rather imported or historical infections. In West Africa a series of xenomonitoring studies have supported this by demonstrating that the predominant mosquito in urban settings (*Culex* spp) is an inefficient vector of *W. bancrofti* and that transmission could not be sustained locally [11,15,16]. Due to the population size and heterogeneity and challenges and tailored strategies to implement effective MDA in urban environments inclusion of such areas unnecessarily represents a significant cost to programmes [17].

In order to facilitate more refined and targeted treatment strategies below implementation unit level or to make decisions or results from standard WHO mapping results are uncertain (e.g., a single positive case), confirmatory mapping protocols have been developed and adopted. Evaluation of the protocols in large rural districts of Tanzania and Ethiopia allowed estimated savings of over 9 million USD by avoiding unnecessary treatment [9]. The confirmatory mapping protocols use a cluster sampling of school attending children aged 9–14 whose infections are more likely (than adults) to be recently acquired but have had a longer period (than the 6–7 year old targeted in TAS) to acquire the infection. MDA decision rules are based on a critical cut off number of children from the sample testing positive that corresponds with an antigenic prevalence of ≥2% in 9–14 year olds [9].

Another proposed approach to confirming the presence or absence of local transmission is the use of entomological methods where vector and sometimes non-vector species that have blood fed can be collected and assessed by qualitative polymerase chain reaction (qPCR) for the presence of parasite DNA which would indicate localized transmission is occurring. Such XM can support monitoring the endgame progress of LF control and elimination activities. [18,19]. Studies in Togo and Ghana have demonstrated the feasibility of using XM for the validation of the absence of LF in previously endemic districts [20,21] and in some cases XM has proven to be more sensitive than serosurveys [22]. At the time of this research, the authors were not aware of any recommended protocol by which to formally undertake XM surveys specifically for confirming transmission prior to beginning treatment in an area of previously unknown endemicity.

Liberia, a sub-Saharan nation in West Africa seeks to eliminate LF by 2030 in line with the WHO roadmap for elimination of NTDs [23]. Mapping was completed in 2010 with LF found to be endemic in 13 of the 15 counties – including Montserrado where the nation capital, Monrovia is located. In 2011, a XM study conducted in 8 communities in Monrovia collected and tested over 3000 mosquitos in pools and found none positive for parasite DNA [15]. MDA commenced in 2012 though notably excluding the two non-endemic counties and Monrovia where there was insufficient data confirming local transmission in the face of resource limitations and well define urban treatment strategy. In 2018 with all countries having received 4–5 rounds of effective coverage and sentinel surveillance indicating reduced MF in almost all counties pre-TAS surveys were begun. By 2020, 12 counties had done pre-TAS with 7 eligible to go on to TAS [24,25]. Montserrado, was the only county where by 2019 sentinel surveillance did not indicate readiness for pre-TAS, and the Ministry of Health therefore sought to establish definitively if there was any need to treat within Monrovia.

This study applied the confirmatory mapping approach to assess if each of the 4 health districts within Monrovia urban area met the WHO antigenic prevalence threshold for beginning MDA. In addition, the study piloted a protocol for the use of XM to inform the same treatment decision. In this paper we present the results of the confirmatory mapping and corresponding recommendations for MDA as well as discuss the potential role of XM in similar urban settings.

## Methods

### Ethics statement

Ethical approval of the study was obtained from the Office of the Institutional Review Board, of the University of Liberia (FWA00004982). For FTS, written informed consent was obtained from parents or guardians of all participants, with children providing individual assent. Participant data were de-identified before importing into STATA statistical software for analysis. All FTS positive cases were reported to health authorities for treatment with ivermectin and albendazole. For mosquito trapping, household head written informed consents were obtained prior to fixing traps. Following that verbal assent was sought each time a trap was emptied, removed or reassembled at their property. Community and school consent was obtained during prior community and school engagement meetings during which the purpose, the procedure and community or school support were discussed.

### Overview

The study employed a structured school based serological survey and XM approach. The serological survey was designed to independently verify transmission and, consequently the need for treatment. XM was included in two of the four health districts to assess it as an alternative decision-making tool.

### Study sites

This study was carried out Monrovia located in Montserrado county of Liberia. Montserrado County was identified as LF endemic in 2010, with a CFA prevalence of 9% [25]. While surrounding peri-urban and rural districts received 4–5 rounds of mass drug administration (MDA) with ivermectin and albendazole through school and community platforms, Monrovia city itself has never been treated, and no sentinel sites have been established. Montserrado county is home to over a third of Liberia’s total population [26] and estimates from the 2022 census put the population of Monrovia itself over 1.5 million [27]. The NTD programme at the MoH who implement MDA in Liberia consider Monrovia in four separate health districts: Central Monrovia in the South, Bushrod to the North East, Somalia Drive to the North and Commonwealth, the largest, to the East. All but Somalia Drive border the Atlantic Coast and all but Central Monrovia are also contiguous with Montessarado County more widely. The city of Monrovia is on the delta of the Mesurado River which bisects the capital resulting in a swampy uninhabited area in the middle that borders all four health districts. Monrovia experiences two distinct rainy seasons, corresponding to two potential LF transmission periods. The short rainy season, occurring between October and November, is followed by the long rainy season, from April to July, during which intense rainfall leads to large-scale flooding and the proliferation of mosquito breeding sites across the city. The annual rainfall is about 1,900 mm and December to April is considered the dry season though it can still rain during this period. *Anopheles gambiae* is the main malaria vector in Monrovia and malaria is endemic throughout the year [28].

### Serological survey

A school-based serological survey was conducted from November 2019 to March 2020 in all four health districts of Montserrado County Central Monrovia, Somalia Drive, Commonwealth, and Bushrod Island following a confirmatory mapping protocol previously described by Gass et al. [9]. School lists provided by the Ministry of Education were mapped to the four health districts to create a sample frame. A total of 30 schools per district were selected using a probability proportionate to size (PPS) selection method. In Central Monrovia that had less than 40 schools all schools were included.

According to Liberia official school age, grades (classes) 3–8 were targeted to find children with the required aged range. A trained team made of laboratory technician and ministry of health staff visited each school and invited children aged 9–14 years who were attending school to participate. All children who presented parental/guardian written consent the next day and individually assented were selected. Each child was registered and demographic data including age, sex, school grade, city residence and travel history were collected. Each participant was assigned a unique QR code, linking their information to a corresponding blood sample and test result.

Each child provided 160 µL of whole blood, obtained via finger prick after cleaning the site with an alcohol swab. Of this, 100 µL was used to detect circulating filarial antigen using Filariasis Test Strips (FTS), while 60 µL was absorbed onto filter paper and stored for future laboratory analysis. The FTS test was performed according to the manufacturer’s protocol, wherein 100 µL of the sample was applied to the absorbent pad of the test strip, and the result was read after 10 minutes. Tests were classified as strong positive, weak positive, negative and invalid according to manufacturer instructions. Positive tests were immediately confirmed with a second test. In the case of negative second test, a third test was completed. Invalid tests were repeated to obtain a valid result. Each test was photographed and later had a second reader verification. Children were provided with the result after ten minutes and those with a verified positive test were later sought out for treatment with ivermectin and albendazole by the Ministry of Health.

Data were collected in the field using an electronic form loaded in the CommCare (Dimagi; https://dimagi.com/commcare/) app, installed on Android smartphones. The data were then downloaded, imported into the STATA statistical package, version 13.0 (TX: StataCorp LP), cleaned, and analysed. Seroprevalence was computed per district as a proportion of the total children tested. Since schools were selected using probability proportionate to size (PPS), they were self-weighting. To account for potential clustering of cases within schools, we aimed for a minimum sample size of 480 per district (350 in Central Monrovia), as determined by Gass et al. [9] for 35% power an incorporating a design effect of 1.5.

### Molecular xenomonitoring

The XM was conducted in Bushrod and Commonwealth health districts as an alternative measure of LF transmission. It was limited to two districts due to financial, logistical, and time constraints. The selection of these districts and communities within was based on their higher malaria endemicity and denser vegetation, identified using the normalized difference vegetation index (NDVI, https://www.usgs.gov/landsat-missions). These were considered proxies of the presence of Anopheles mosquitoes which are efficient LF vectors in Africa [28].

Mosquito collection took place between May and October 2019. Eight communities were selected per district, aiming to collect at least 10,000 mosquitoes, including 1,500 *Anopheles* per district, using a combination of gravid (GT) and exit traps (ET). Each community had six exit traps (ETs) and one gravid trap (GT) with ETs operated for 5 days per month in households and the GT for four days rotating between 4 outdoor collection sites. Number and configuration of traps to achieve the sample size was initially based on Nditanchou *et al*. [29] and trapping outputs were periodically reviewed and adjusted to promote larger sample size.

Communities were sensitized, and consent was obtained through their leaders. Two trained community entomologists and a local guide were assigned per community. Household selection followed a predetermined sampling interval based on the number of houses and ETs. Only consenting households were recruited. Traps were emptied daily in the mornings using manual aspirator tubes. In addition, the collection team noted local weather conditions, trap state and human activity and their potential impact on mosquito trapping efficiency.

Each morning, the mosquitoes were put in clean plastic cups sealed with pieces of mosquito net secured with a rubber band and transported to the laboratory. Any live mosquitos were knocked down by adding acetone-soaked cotton wool to the cups and they were sorted by sex and abdominal condition (unfed, blood-fed, semi-gravid, gravid) by trained entomologists. Male mosquitoes were discarded, while females were further sorted into species categories (*Anopheles gambiae*, other anophelines, *Culex*, and all other species) using a simplified taxonomic key and counted [30,31].

### *Wuchereria bancrofti* DNA detection in mosquitoes

Mosquitoes were packaged with desiccant, labelled according to collection site, species category, and abdominal status, and transported to Liverpool School of Tropical Medicine for qPCR assay to detect L3 *W. bancrofti* larvae. All *An. gambiae* s.l. and *Culex spp.* mosquitoes were grouped into pools, with a maximum of 10 mosquitoes per pool for *An. gambiae* s.l., and 30 per pool for *Culex*. In total, 302 pools of *An. gambiae* s.l. and 545 pools of *Culex* were analyzed by qPCR assay.

### DNA extraction

DNA extraction from mosquito carcasses was performed using Qiagen DNeasy Blood & Tissue Kits (Qiagen, Manchester, UK) following standard protocols including a mechanical disruption stage using a TissueLyser bead mill (Qiagen). Pooled samples were placed into twelve 1.2 mL 8 strip tubes in a rack, each well containing a ball bearing and 200 µL of ATL Buffer and Proteinase K mix. Samples were crushed in the Tissue Lyser at 30 1/S frequency for five minutes, then incubated overnight at 56°C. After incubation they were centrifuged at 2,000 rpm followed by addition of 410 µL of AL buffer/ ethanol mix. Lysates were transferred to a DNeasy 96 plate on top of an S-block and centrifuged for 15 min. at 6,000 rpm. Subsequent buffer washes were performed: 500 µL of buffer AL1 was added and centrifuged for 5 min at 6,000 rpm, followed by 500 µL of buffer AL2 with another 15 min centrifugation at 6,000 rpm. The final elution step involved adding 100 µL of AE buffer to each well and centrifuging for 3 min. at 6,000 rpm and then repeating the process, yielding a final DNA solution of 200 µL.

DNA amplification was conducted using a SYBR qPCR assay targeting the long direct repeat DNA sequence of *W. bancrofti* [32]. Each reaction mixture contained 2 µL of template DNA, 0.5 µL of each primer, 12.5 µL of iTaq SYBR Green buffer and 9.5 µL of nuclease-free water, with positive and negative controls included for quality control. qPCR involved an initial denaturation cycle of 3 min. at 95°C, followed by 40 cycles of 10 seconds at 95°C and 1 min. at 60°C. After amplification a final temperature gradient between 55°C and 95°C was applied to generate a dissociation curve allowing for parasite detection and verification.

Confirmatory testing was performed for samples with ambiguous dissociation curves using a probe-based qPCR assay targeting the *W. bancrofti* Tandem Repeat 1, following the methods reported by Zulch et al. [33]. Reactions included 2.5 µL of template DNA mixed with a Master Mix comprising 0.3125 µL of forward primer, 2.5 µL of reverse primer and 0.622 µL of probe. PCR conditions included an initial hold at 50°C for 2 minutes then 95°C for 10 minutes, followed by 45 cycles of 15 seconds at 95°C and 1 minute at 60°C.

All field data were recorded electronically via CommCare on Android smartphones, while mosquito infection was calculated using the Poolscreen v2.0 to determine the maximum likelihood of infection together with the associated 95% Confidence Interval, CI [34].

## Results

### Serology sampling

Of the selected 119 schools 6 transpired to be in different health districts to where they were mapped. Nineteen schools (including 10 in Commonwealth) had to be replaced because they had closed and/or could not be located by the team. In Central Monrovia, there was no replacement because sampling was exhaustive of the list, and 3 schools were identified by the team that were not on the list. Overall, 20% of the sample of school children came from replacement or previously unidentified schools. Finally, after schools were reallocated to their correct districts, 106 schools had been sampled and both Somalia Drive and Bushrod were short of 4 clusters from the initial target (Table 1).

**Table 1:** School and children sampling outcome.

Health District	Estimated enrolled 9–14 yr olds	# schools	Sampling Strategy^1^	Schools selected	Cluster size range	Target Sample Size^1^	Replaced Schools	Schools Sampled	Children tested	children from replacement schools (%)
**CW**	5,918	454	Cluster PPS	30	4-45	480	10	34	579	23.5
**SD**	7,772	310	Cluster PPS	30	1-48	480	5	26	443	23.3
**BR**	7,652	272	Cluster PPS	30	2-34	480	4	26	411	12.7
**CM**	6,234	29	Exhaustive	29	7-39	320	3*	20	366	17.8
**TOTAL**	**27,577**	**1065**		**119**	**1-48**	**1760**	**19**	**106**	**1799**	**19.8**

CW: Commonwealth, SD: Somalia Drive, BR: Bushrod Island, CM: Central Monrovia PPS: Probability Proportional Size.

^1^As determined by the confirmatory mapping protocols [8], *Schools identified not on the sampling list.

Within schools, children were found to be often in a higher or lower grade than expected and low rates of parental consent return from selected children led to small clusters. From day four of the survey the team therefore used convenience sampling of children identified in the target age group as described in the methods; this is a modification of the protocol that specifies the use of a sampling fraction or systematic sample and targeting classes corresponding to age group. The target sample size of children was met in Central Monrovia and Commonwealth.

### Sample characteristics

Blood samples were obtained for 1,852 school children. In 53 cases, there was insufficient blood for a valid test and these children were excluded from the sample. 1799 children aged 9–14 in 106 schools were tested. In each health district females were in the slight majority. Seventy eight percent of children reported being born in Monrovia, while 33.7% reported they may have been exposed to an LF endemic area having previously lived or travelled for over a month or having received MDA at a past point. The mean age of the sample was 11 (Table 2).

**Table 2:** Sample characteristics per health district.

Health District	Children (#)	Female (%)	Age Range	Mean age	Born in Monrovia (%)	Lived in endemic area (%)	Travelled to endemic area (%)	MDA participation (%)	Any endemic exposure (%)	Slept under bed net previous night (%)
**CW**	579	53%	9-14	11.7	70.3	29.0	24.4	9.5	50.6	40.0
**SD**	443	54%	9-14	11.5	87.1	12.6	0.5	8.4	20.5	35.0
**BR**	411	57%	9-14	11.3	83.5	15.3	5.6	4.4	22.9	26.8
**CM**	366	64%	9-14	11.1	74.0	25.4	17.2	0.6	35.3	24.3
**Total**	**1799**	**56.4%**	**9-14**	**11.4**	**78.2**	**21.2**	**12.7**	**6.2**	**33.7**	**32.5**

CW: Commonwealth, SD: Somalia Drive, BR: Bushrod Island, CM: Central Monrovia.

### FTS tests

Overall, 96 children in 32 schools tested positive and there were 3 or more positive cases in every health district. Each health district therefore exceeded the critical cut off of 3 positive cases, equivalent to CFA ≥ 2% in 9–14 year olds [9] (Table 3).

**Table 3:** Results of the FTS tests.

Health District	Children tested	Positive Tests	# Schools Positive	Antigenic Prevalence % (CI)	Critical Cut Off^1^	Result^1^	Outcome^1^
**CW**	579	19	4	3.3 (2.1-5.1)	3	Fail	Begin MDA
**SD**	443	59	17	13.3 (10.3-16.8)	3	Fail	Begin MDA
**BR**	411	14	9	3.4 (1.9-5.6)	3	Fail	Begin MDA
**CM**	366	4	2	1.1 (0.3-2.8)	3	Fail	Begin MDA
**TOTAL**	**1,799**	**96**	**32**	**5.3 (4.4 -6.5)**			

CW: Commonwealth, SD: Somalia Drive, BR: Bushrod Island, CM: Central Monrovia.

^1^As determined by the confirmatory mapping protocols [8].

**Fig 1:**
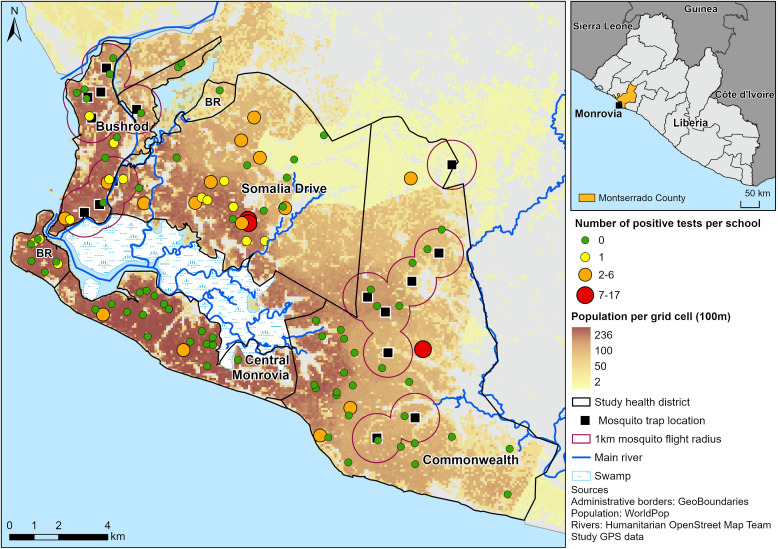
Locations of sampled schools and mosquito collection sites and the number of positive tests per school.

**Fig 2:**
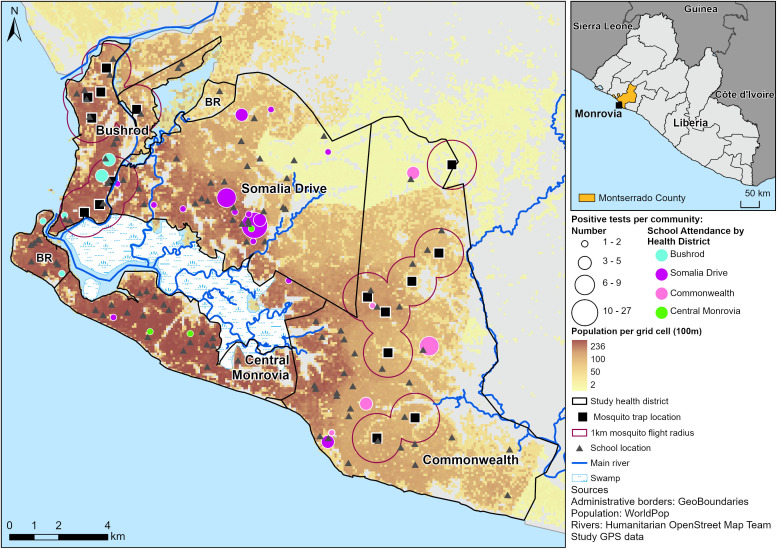
Community of residence and health district of school attendance for all positive tests. **NB:** 7 of 96 positive cases could not be mapped to the community of residence Bushrod (4) Somalia Drive (2) Commonwealth (1) Central Monrovia (0).

Somalia Drive had the highest antigenic prevalence 13.3% (10.3-16.8%) with 59 positive tests in 17 of the 26 schools sampled. Seven of the schools had only a single positive test, while one school had 17 positive tests from 29 children. (Fig 1, Table 3). Though there were 17 positive clusters in Somalia Drive, mapping cases back to the community of residence of the children revealed that they all lived within 11 communities, 3 of which (Chicken Soup Factory, Oxygen Factory and Gardnerville) bordered each other and accounted for 36 (61%) of the Health District’s positive cases (Fig 2). Six children who tested positive lived in Commonwealth and one in Bushrod in communities close to positive clusters there. One child who tested positive lived in Central Monrovia. Thirty-four percent of the children testing positive indicated that they may have had some endemic exposure through somewhere outside Monrovia that they either lived or travelled earlier in their lives (Table 4).

**Table 4:** Characteristics of positive tests.

Health District	Children (#)	Female (%)	Age Range	Mean age	Born in Monrovia (%)	Lived in endemic area (%)	Travelled to endemic area (%)	MDA participation (%)	Any endemic exposure (%)	Live outside Schooling District^1^ # (%)
**CW**	19	57.9	9-14	11.8	52.6	47.4	21.1	0.0	57.9	0 (0)
**SD**	59	54.2	9-14	11.9	81.4	17.0	0.0	17.0	33.9	7 (12)
**BR**	14	42.9	9-14	11.1	71.4	21.4	0.0	0.0	21.4	0 (0)
**CM**	4	25.0	10-14	11.8	75.0	25.0	0.0	0.0	25.0	2 (50)
**Total**	**96**	**52.1**	**9-14**	**11.8**	**74.0**	**24.0**	**4.2**	**10.4**	**36.5**	**9 (10)**

CW: Commonwealth, SD: Somalia Drive, BR: Bushrod Island, CM: Central Monrovia.

^1^N = 89 as 7 positive tests could not be mapped to their place of residence (SD (2), CW (1), BR (4)).

In Central Monrovia, the antigenic prevalence was 1.1% with a confidence interval that crossed the 2% threshold. There were 4 positive tests in 2 schools with each school having 2 positives. (**Fig 1**, **Table 3**). Two of the children testing positive in Central Monrovia lived in Chicken Soup Factory in Somalia Drive and one child testing positive in a school in Somalia Drive lived in Central Monrovia (Fig 2). Only one child testing positive had a travel history suggesting endemic exposure and this was one of the children living in Somalia Drive.

In Bushrod, 14 children were positive from 9 schools (Fig 1, Table 3) and antigenic prevalence was 3.4% (1.9-5.6%). When mapping positive cases to their communities of residence 4 could not be mapped. Of the remaining 10, 7 lived in close proximity in a group of three communities in close proximity and near to the border with Somalia Drive. (Fig 2).

No cases were mapped as living outside of Bushrod (though 4 were unmapped) and 1 child testing positive from a school in Somalia Drive was confirmed to live in Bushrod. The travel history of 21% of these positive testing children gave an indication of some previous endemic exposure to LF (Table 4).

In Commonwealth, antigenic prevalence was 3.3% (2.1-5.1%) with 19 positive tests coming from 4 schools (Fig 1, Table 3). With the exception of two - one of which that could not be mapped, all the children testing positive in Commonwealth reported residing there in four communities close to their schools (GSA Road, Konka Town, King Gray and Soul Clinic). Nine of the 19 positive tests lived in Soul Clinic. Five children who tested positive in schools in Somalia Drive also mapped back to two other communities in Commonwealth – Bassa Town and Stephen Tolbert Estate. Fifty eight percent of children reported previously living or substantial travelling to an LF endemic area (Table 4).

Figs 1 and 2 were created using ArcGIS PRO software by Esri. Administrative boundaries were from GeoBoundaries (https://www.geoboundaries.org) [35]), population counts from WorldPop (www.worldpop.org) [36], and Rivers from Humanitarian OpenStreetMap Team (https://data.humdata.org/dataset/hotosm_lbr_waterways).

### Mosquito collections

Overall, 9240 female mosquitos, 1,080 *An. gambiae*, 7,667 *Culex* and 472 mosquitos of other species or genus were collected in Bushrod District (Table 5). In Commonwealth, a total sample of 10,869 female mosquitos was comprised 1,940 *An. gambiae*, 8,668 *Culex* and 260 other mosquitos (Table 5). In both districts, 25% of the mosquitos had had a recent bloodmeal being classified as fed, semi-gravid or gravid.

**Table 5:** Female mosquitos collected in each district and overall by species and blood fed status.

District	Species	Unfed n	Fed n	Semi-gravid n	Gravid n	Total n	blood meal^1^%
**BR**	*Culex*	5613	78	642	1334	7667	26.8
	*An. gambiae*	896	101	21	62	1080	17.0
	Other	381	85	7	20	493	22.7
	**Total**	6890	264	670	1416	9240	25.4
**CW**	*Culex*	6457	262	576	1373	8668	25.5
	*An. gambiae*	1474	350	35	81	1940	24.0
	Other	244	13	4	0	261	6.5
	**Total**	8175	625	615	1454	10869	24.8
**Total**	*Culex*	12070	340	1218	2707	16335	26.1
	*An. gambiae*	2370	451	56	143	3042	21.5
	Other	625	98	11	20	754	17.1
	**Total**	15065	889	1285	2870	20109	25.1

CW: Commonwealth, BR: Bushrod, ^1^fed+semi gravid + gravid

**Fig 3:**
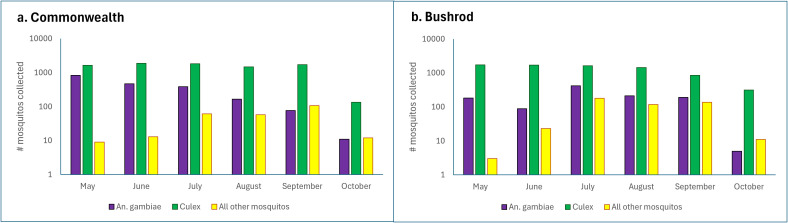
A and B) Total number of *Anopheles* and *Culex* caught per month in Commonwealth and Bushrod Districts.

S1A and S1B Table shows catches per community for gravid traps and exit traps. In Bushrod, the number of *An. gambiae* collected was highest in May and declined gradually until October. In Commonwealth, and for *Culex* in Bushrod mosquitos there was similarly an overall decline from start to finish but the numbers collected initially rose from May and peaked in July before declining until October (Fig 3A and 3B) collections of mosquitos over time in two health districts.

### Trap performance

Overall, 3,631 trapping events were undertaken. Of these, 3,226 were ETs and 405 were GTs. A total of 19,355 potential vectors of *W. bancrofti* were collected, of which 10,029 *Culex* and 2,899 *An. gambiae* were recorded in ETs, giving a mean of 4.0 mosquitoes per ET trapping event (3.1 *Culex* and 0.9 *An. gambiae*). Recently fed mosquitoes represented 19%, corresponding to a mean catch of 0.78 recently fed mosquitoes per ET trapping event.

For the GTs, 6,306 *Culex* and 121 *An. gambiae* were collected, with a mean of 16.6 mosquitoes (15.6 *Culex* and 1.0 *An. gambiae*) caught. Thirty eight percent of *Culex* and 11% of *An. gambiae* were recently fed per trapping event corresponding to a catch of 5.95 recently fed mosquitos per gravid trap event.

### Detection of *Wuchereria bancrofti* DNA in mosquitoes

In both health districts, all the potential vector mosquitoes were selected and analyzed to determine the infection rate and no infected mosquitoes were detected from the communities (Table 6).

**Table 6:** Proportion of positive mosquitoes for *Wuchereria bancrofti* DNA.

	*An. gambiae* s.l.				*Culex* spp			
District	Mosquitos #	Pools #	PCR positive pools	Infection rate % (95%CI)	Mosquitos #	Pools #	PCR positive pools	Infection rate % (95%CI)
BR	1,080	108	0	0 (0.00-0.18)	7,667	256	0	0 (0.00-0.03)
CW	1,940	194	0	0 (0.00-0.09)	8,668	289	0	0 (0.00-0.02)
**Total**	3,020	302	0	0 (0.00-0.06)	16,335	545	0	0 (0.00-0.01)

BR: Bushrod, CW: Commonwealth, CI: Confidence Interval, NB: No infection was found in mosquitos collected.

## Discussion

Liberia has made significant progress towards LF elimination with 12 of 13 endemic counties undertaking pre-TAS and several passing and moving to TAS [24]. Sentinel surveillance in Montserrado however has not indicated the same declines in MF and in 2019 the Ministry of Health sought to confirm if Monrovia City should be included to the ongoing MDA in this county.

Following the confirmatory mapping protocols recommended by WHO [37], in which children aged 9–14 were tested for CFA using FTS, more than 3 children tested positive per health district - the critical threshold indicating that districts require MDA [9]. In Somalia Drive, Bushrod, Commonwealth and Central Monrovia the numbers of children testing positive were 59, 14, 19, and 4 corresponding to antigenic (CFA) prevalence of 13.3% (95% CI: 10.3-16.8), 3.4% (1.9-5.6), 3.3% (2.1-5.1), 1% (0.3-2.8), respectively. Apart from Central Monrovia, these indicate high antigenicity calling for immediate initiation of MDA in all the health districts.

Confirmatory mapping protocols are recommended for low prevalence or perceived non-endemic areas where the traditional mapping approach is not specific enough [9]. In such contexts it is rare to detect such high rates of positive cases, the highest identified in the literature was in India, confirmatory mapping of two non-MDA districts both of which met the critical cut off with 27 and 8 positive cases identified from 480 children in each district [38]. In other published results, the confirmatory mapping tool returned lower rates and had few districts meeting the critical cut off. In Zimbabwe, confirmatory mapping in 39 districts previously categorised endemic returned only 79 positives from over 18,000 children and only 7 districts exceeded the critical cut off [39]. In Ethiopia, 29 cases (0.16%) from 45 woredas (districts) with only 3 woredas meeting the critical cut off [40] and in Tanzania 1 positive case in 10 districts with no districts reaching the critical cut off [9].

One potential explanation for the high number of positive tests is population movement from surrounding areas that are LF endemic. This movement might have brought in active infections with MF and/or adult worms as well as recent past infections that would still record positive for CFA. Liberia has been steadily urbanising and in 2020 recorded urban population growth of 3% compared to 2.1% population growth nationally [41]. During the second civil war (1993–2003) over 500,000 people were internally displaced, many of whom relocated to Monrovia, the population of which was estimated to have tripled at the peak of the conflict [42]. Many of these people resettled permanently in Monrovia and the population has continued to rise, estimated at 1 million in 2009 and 1.5 million by 2020 (https://population.un.org/wpp/).

It is important to also consider that CFA is a measure of presence or recent presence of adult worms and not definitive of MF which are required along with a sufficiently efficient vector mosquito for transmission to be occurring. While this is accounted for in the confirmatory mapping decision table with the critical cut off being based on WHOs selection of 2% antigenemia prevalence corresponding to 1% MF prevalence [43], this estimate is very conservative favouring a higher MF estimate and more MDA. A recent study in India [44], in a post MDA setting found that MF prevalence among 5–17 year olds was about a third of antigenic prevalence detected by FTS. A study in pre-MDA settings including Liberia found that of 302 positive FTS tests 97 (32%) were confirmed MF positive [45]. In Tanzania in an area of previously high endemicity after 15 years plus of MDA a 5.8% CFA was recorded of which only 5.2% were MF positive [46]. In a pre-MDA context such as Monrovia with incoming migration from endemic areas under MDA, the relationship between CFA, MF and transmission could be similarly about a third of CFA, or potentially further skewed.

Historically, MF cases identified in Monovia were attributed to being transient individuals from the provinces [47]. In this study, there was little evidence that a majority of positive tests could be explained as imported or past infections. With the survey targeting 9–14 year olds, any adult worm infection reflected by the CFA must have been relatively recent with that age group to young to have been imported during the mass population movement of the civil war. Of the 96 positive testing children less than 40% (n = 35) indicated any potential route of importing specifically that they had either lived in an endemic district, travelled to one for more than a month or had been part of MDA at an earlier. Also of consideration is that all endemic districts have been under effective MDA since 2012, successfully reducing the MF prevalence. This would mean that for the immediately preceding years, exposure to transmission during travel to and residence in these areas would have been much reduced.

A second possible explanation could be diagnostic or technician performance. However we have no reason to support such a theory. The DNA extracts from the mosquitos were processed using a robust *W. bancrofti* WbTR1 qPCR protocol with appropriate quality control as detailed in the methods. The spread of positive FTS tests was across batches of tests, FTS being a stable test that was stored, administered and read according to manufacturers instructions with all positive tests confirmed with a second positive test and all tests double read. Liberia is not co-endemic for loa loa [48] and so the known cross reactivity with loiasis can also be excluded [49,50].

XM is used as a complimentary strategy to TAS in post elimination monitoring for recrudescence [22,37,51], and has been identified as a more sensitive indicator than either MF or CFA in low prevalence settings [37,52,53]. A threshold of <0.25% infection with *W.bancrofti* DNA among gravid mosquitos is often applied to indicate that transmission is unsustainable [54]. In this study, *Culex*, a less efficient vector than the *An. gambiae* complex were identified as the dominant mosquito. This is in line with the literature that highlights species of the *Cx*. *pipiens* complex are widespread and predominant in urban environments in Africa and that in particualar *Cx*. *quinquefasciatus* is dominant in West African cities [54]. XM in the two health districts (Commonwealth, Bushrod) did not identify any pools of mosquitos positive for *W. bancrofti* DNA. Although this is in agreement with the findings of de Souza et al [28], it is discordant with serology in the study which gave a strong signal of transmission in both health districts. A significant contributing factor in this ‘failure’ of XM to identify transmission is likely to be the lack of geographical overlap between XM and serology as illustrated in Figs 1 and 2. This is especially pertinent given the low flight range of *Cx. quinquefasciatus* and *An. gambiae* that both average less than a km with the range for weak flyers generally estimated as low as 50 metres in an urban landscape. Mosquitos were not collected in the same areas as serology was conducted and consequently no mosquitos were collected in areas where positive tests were identified and transmission suspected. This is an unfortunate outcome of the sampling strategy applied, which in the absence of school data or any preexisting prevalence data, was based on targeting areas where vector mosquitos were likely to be due to concerns over sample size. Sample size itself could also be a further contributing factor since blood fed mosquitos of epidemiological interest did not exceed 2700 in either Health District and gravid mosquitos specifically did not exceed 1500. These are low compared to many examples in the literature though required more trapping events and were also lower on average than those recorded by Nditanchou et al. [29] in a similar *Culex* dominated West African urban environment of Kaduna, Nigeria.

In summary, even considering a less conservative link between antigenemic and MF prevalence than that underpinning the confirmatory mapping protocols the weight of evidence supports the serological indication of local transmission occurring over the XM indication that it is not. Taking all of the above into account the recommendation to begin MDA in Somalia Drive, Bushrod and Commonwealth based on the confirmatory mapping decision table is clear.

In Central Monrovia, the recommendation is less clear as the number of cases was just above the critical threshold. Furthermore, two of the four positive cases in Central Monrovia live in Somalia Drive Health District and specifically in Chicken Soup Factory – a community where 27 other positive tests (almost a third of all positive tests identified) also confirmed residing. A final consideration is that the confirmatory mapping protocols were developed with ICT cards whereas they are now being implemented – and were in this case – with FTS which are more sensitive and return a higher number of positives [45]. As Central Monrovia is the most populated district of Monrovia, and therefore a more challenging and costly place to implement MDA, a small study to further confirm transmission would be advisable.

As well as making the interpretation of the treatment recommendation in Central Monrovia less clear, this local importation of cases highlights an issue of geographical representativeness of a school-based survey in a setting with a large choice of schools and where children may travel a considerable distance – potentially cross district- to attend.

The confirmatory mapping protocol, designed for larger rural low prevalence districts with school enrolment over 75% and are consciously conservative – leaning towards favouring MDA over not and based on use of ICT [9]. In this study we have attempted to implement them in an urban area with low school enrolment and where school and area of residence do not necessarily overlap. As detailed in the results we also encountered a series of logistical challenges with the sampling strategy stemming from the urban environment. In particular: large school lists were hard to map to health implementation units and proved to be out of date with a high proportion of private schools that reportedly opened and closed with regularity. Limited feasibility to engage effectively with parents led to low rates of consent and therefore participation making it hard to achieve the sample size and representativeness and leading to very uneven clusters. The serology survey adopted convenience sampling in schools in place of a sampling fraction or systematic sampling. These contextual differences and methodological challenges potentially undermine the statistical basis of the sampling strategy and modelled relationships between critical cut off, antigenic prevalence and MF prevalence. An adapted urban confirmatory mapping protocol may be a useful addition to the set of tools for programme managers. Such a protocol could consider a critical cut off that reflected the possibility of imported cases (including through cross district school attendance); include additional sampling strategies and the conditions that would merit using them and could provide suggested transmission validation options (including XM) where the scale of MDA under consideration warrants an extra degree of certainty close to the thresholds. Further research into the relationship between CFA and MF in different contexts – especially urban – and the impacts of ineffective vector dominance could better determine if the critical cut off could be based on a less conservative MF threshold in some settings. Research into the feasibility and cost effectiveness of implementing MDA at a subunit level (for example ward) could also be considered.

XM in support of LF elimination has mainly been conducted in research settings and gaps remain as to survey design and interpretation of results, and the relationship between infection in mosquitos and MF and CFA in humans [22]. Discordant results at cluster level are common, sometimes even when data is collected in the same household [23,37]. In one of the few other studies investigating XM as a method of informing the ‘start MDA Panda et al. [36]’ decision implemented XM alongside the confirmatory mapping protocols and a community-based MF survey. They used the same 30 PPS selected clusters and systematically selected a total of 150 HH in the district to host gravid traps. Their sampling strategy could be regarded as successful in that they had district level agreement between XM and CFA and is also more closely aligned to other XM studies which similarly took a cluster based representative approach [20,22,51,54]. This sample size and approach could be built upon, adapted and validated for urban areas where mosquito catches are lower, and there are no sparsely or unpopulated areas between clusters as there are in rural districts. As a less intrusive measure of transmission with potential for higher sensitivity in low prevalence areas XM could complement confirmatory mapping in urban environments especially where confirmatory mapping outcomes are uncertain. Further research including modelling of different sampling strategies, construction of more evidence based thresholds and detailed cost effectiveness analysis are required.

### Limitations

The major limitation in the study is the selection of XM districts and sites separately from and prior to serology selections. As a result, there is poor geographical overlap between the two methods leading to inability to look at agreement or discordance between methods at a cluster level and minimising the utility of the XM. Challenges implementing the confirmatory mapping protocol sampling strategy meant only two of the four health districts reached the targeted sample size. Since the remaining two districts both exceeded the critical cutoff by a large margin we do not believe this invalidates the findings. Finally, this study did not confirm CFA positivity with night smears to establish MF positivity. While not a requirement for the confirmatory mapping protocols it would have been valuable to elucidate the relationship between CFA and MF in this context.

### Conclusion

Mass drug administration is recommended for implementation in Bushrod, Somalia Drive, and Commonwealth. In Central Monrovia, the prevalence is near the threshold, with several cases likely attributable to importation. Therefore, further confirmatory activities are advised prior to MDA initiation. The sampling strategy employed for XM in this setting was not optimal, however, with appropriate adaptations, XM could serve as a valuable supplementary tool for validating borderline results in confirmatory mapping protocols, particularly in urban contexts. The development and integration of an adapted confirmatory mapping protocol tailored for urban environments would significantly enhance the suite of tools available to programme managers, thereby improving intervention precision and disease control strategies.

## Supporting information

S1 Table A) Community catches for Exit Traps, B) Community catches for Gravid Traps. (DOCX)

## References

[1] World Health Organization. Lymphatic filariasis: status of mass drug administration. 2024. https://apps.who.int/neglected_diseases/ntddata/lf/lf.html

[2] World Health Organization. Global programme to eliminate lymphatic filariasis: progress report, 2022. Weekly Epidemiological Record. 2023;41(98):489–502.

[3] World Health Organization. Guideline: alternative mass drug administration regimens to eliminate lymphatic filariasis. 2017. 29565523

[4] World Health Organization. Preventive chemotherapy in human helminthiasis. Geneva: 2006.

[5] World Health Organization. Lymphatic filariasis: practical entomology. A handbook for national elimination programmes. Geneva: World Health Organization; 2013.

[6] Irvine MA, Stolk WA, Smith ME, Subramanian S, Singh BK, Weil GJ, et al. Effectiveness of a triple-drug regimen for global elimination of lymphatic filariasis: a modelling study. Lancet Infect Dis. 2017;17(4):451–8. doi: 10.1016/S1473-3099(16)30467-4 28012943

[7] Maclean MJ, Lorenz WW, Dzimianski MT, Anna C, Moorhead AR, Reaves BJ, et al. Effects of diethylcarbamazine and ivermectin treatment on Brugia malayi gene expression in infected gerbils (Meriones unguiculatus). Parasitol Open. 2019;5:e2. doi: 10.1017/pao.2019.1 33777408 PMC7994942

[8] World Health Organization. Lymphatic filariasis: monitoring and epidemiological assessment of mass drug administration. World Health Organization; 2011. https://www.who.int/publications/i/item/9789241501484

[9] Gass KM, Sime H, Mwingira UJ, Nshala A, Chikawe M, Pelletreau S, et al. The rationale and cost-effectiveness of a confirmatory mapping tool for lymphatic filariasis: examples from Ethiopia and Tanzania. PLoS Negl Trop Dis. 2017;11(10):e0005944. doi: 10.1371/journal.pntd.0005944 28976981 PMC5643143

[10] Pam DD, de Souza DK, D’Souza S, Opoku M, Sanda S, Nazaradden I, et al. Is mass drug administration against lymphatic filariasis required in urban settings? The experience in Kano, Nigeria. PLoS Negl Trop Dis. 2017;11(10):e0006004. doi: 10.1371/journal.pntd.0006004 29020042 PMC5665554

[11] Kouassi BL, de Souza DK, Goepogui A, Narh CA, King SA, Mamadou BS, et al. Assessing the presence of Wuchereria bancrofti in vector and human populations from urban communities in Conakry, Guinea. Parasit Vectors. 2015;8:492. doi: 10.1186/s13071-015-1077-x 26410739 PMC4583765

[12] World Health Organization. The expanded special project for elimination of neglected tropical diseases (ESPEN) 2017 annual report. Regional Office for Africa; 2018.

[13] Hast MA, Javel A, Denis E, Barbre K, Rigodon J, Robinson K, et al. Positive-case follow up for lymphatic filariasis after a transmission assessment survey in Haiti. PLoS Negl Trop Dis. 2022;16(2):e0010231. doi: 10.1371/journal.pntd.0010231 35213537 PMC8906642

[14] Koroma JB, Sesay S, Sonnie M, Hodges MH, Sahr F, Zhang Y, et al. Impact of three rounds of mass drug administration on lymphatic filariasis in areas previously treated for onchocerciasis in Sierra Leone. PLoS Negl Trop Dis. 2013;7(6):e2273. doi: 10.1371/journal.pntd.0002273 23785535 PMC3681681

[15] de Souza DK, Sesay S, Moore MG, Ansumana R, Narh CA, Kollie K, et al. No evidence for lymphatic filariasis transmission in big cities affected by conflict related rural-urban migration in Sierra Leone and Liberia. PLoS Negl Trop Dis. 2014;8(2):e2700. doi: 10.1371/journal.pntd.0002700 24516686 PMC3916318

[16] Koudou BG, de Souza DK, Biritwum N-K, Bougma R, Aboulaye M, Elhassan E, et al. Elimination of lymphatic filariasis in west African urban areas: is implementation of mass drug administration necessary?. Lancet Infect Dis. 2018;18(6):e214–20. doi: 10.1016/S1473-3099(18)30069-0 29402636

[17] Adams AM, Vuckovic M, Birch E, Brant TA, Bialek S, Yoon D, et al. Eliminating neglected tropical diseases in urban areas: a review of challenges, strategies and research directions for successful mass drug administration. Trop Med Infect Dis. 2018;3(4):122. doi: 10.3390/tropicalmed3040122 30469342 PMC6306919

[18] Okorie PN, de Souza DK. Prospects, drawbacks and future needs of xenomonitoring for the endpoint evaluation of lymphatic filariasis elimination programs in Africa. Trans R Soc Trop Med Hyg. 2016;110(2):90–7. doi: 10.1093/trstmh/trv104 26822601

[19] Pi-Bansa S, Osei JHN, Joannides J, Woode ME, Agyemang D, Elhassan E, et al. Implementing a community vector collection strategy using xenomonitoring for the endgame of lymphatic filariasis elimination. Parasit Vectors. 2018;11(1):672. doi: 10.1186/s13071-018-3260-3 30587226 PMC6307201

[20] Dorkenoo MA, de Souza DK, Apetogbo Y, Oboussoumi K, Yehadji D, Tchalim M, et al. Molecular xenomonitoring for post-validation surveillance of lymphatic filariasis in Togo: no evidence for active transmission. Parasit Vectors. 2018;11(1):52. doi: 10.1186/s13071-017-2611-9 29361964 PMC5781303

[21] de Souza DK, Otchere J, Ahorlu CS, Adu-Amankwah S, Larbi IA, Dumashie E, et al. Low microfilaremia levels in three districts in coastal ghana with at least 16 years of mass drug administration and persistent transmission of lymphatic filariasis. Trop Med Infect Dis. 2018;3(4):105. doi: 10.3390/tropicalmed3040105 30274501 PMC6306872

[22] McPherson B, Mayfield HJ, McLure A, Gass K, Naseri T, Thomsen R, et al. Evaluating molecular xenomonitoring as a tool for lymphatic filariasis surveillance in Samoa, 2018-2019. Trop Med Infect Dis. 2022;7(8):203. doi: 10.3390/tropicalmed7080203 36006295 PMC9414188

[23] World Health Organization. Ending the neglect to attain the Sustainable Development Goals: a road map for neglected tropical diseases 2021–2030. Geneva: World Health Organization. 2020. https://www.who.int/publications/i/item/9789240010352

[24] Republic of Liberia Ministry of Health. Master plan for neglected tropical diseases 2016-2020. 2016. https://espen.afro.who.int/system/files/content/resources/LIBERIA_NTD_Master_Plan_2016_2020.pdf

[25] Republic of Liberia Ministry of Health. Master Plan for Neglected Tropical Diseases 2023-2027. 2024. https://espen.afro.who.int/tools-resources/documents/country-ntd-master-plans

[26] UNFPA L. Annual report, accelerating the transformative results in liberia. progress opportunities and learnings. 2023. https://liberia.unfpa.org/sites/default/files/pub-pdf/2024-10/UNFPA%20Liberia%20Country%20Office%20%202023%20Annual%20Report.pdf

[27] Liberia Institute of Statistics and GIS. Liberia population and housing census. Final results. 2023. https://lisgis.gov.lr/document/LiberiaCensus2022Report.pdf

[28] de Souza DK, Koudou BG, Bolay FK, Boakye DA, Bockarie MJ. Filling the gap 115 years after ronald ross: the distribution of the *Anopheles coluzzii* and *Anopheles gambiae* s.s from freetown and monrovia, West Africa. PLoS One. 2013;8(5):e64939. doi: 10.1371/journal.pone.0064939 23741429 PMC3669227

[29] Nditanchou R, Dixon R, Pam D, Isiyaku S, Nwosu C, Sanda S, et al. Testing a method of sampling for entomological determination of transmission of *Wuchereria bancrofti* to inform lymphatic filariasis treatment strategy in urban settings. Parasit Vectors. 2020;13(1):37. doi: 10.1186/s13071-020-3905-x 31973747 PMC6979341

[30] Gillies MT, De Meillon B. The Anophelinae of Africa south of the Sahara (Ethiopian zoogeographical region). Johannesburg: South African Institute for Medical Research; 1968.

[31] Harbach R. The mosquitoes of the subgenus Culex in southwestern Asia and Egypt (Diptera: Culicidae). Contributions of the American Entomological Institute. 1988;24(1):247.

[32] Rao RU, Atkinson LJ, Ramzy RMR, Helmy H, Farid HA, Bockarie MJ, et al. A real-time PCR-based assay for detection of *Wuchereria bancrofti* DNA in blood and mosquitoes. Am J Trop Med Hyg. 2006;74(5):826–32. 16687688 PMC2196401

[33] Zulch MF, Pilotte N, Grant JR, Minetti C, Reimer LJ, Williams SA. Selection and exploitation of prevalent, tandemly repeated genomic targets for improved real-time PCR-based detection of *Wuchereria bancrofti* and *Plasmodium falciparum* in mosquitoes. PLOS ONE. 2020;15(5):e0232325. 10.1371/journal.pone.0232325 PMC7194414 32357154

[34] Katholi CR, Toé L, Merriweather A, Unnasch TR. Determining the prevalence of Onchocerca volvulus infection in vector populations by polymerase chain reaction screening of pools of black flies. J Infect Dis. 1995;172(5):1414–7. doi: 10.1093/infdis/172.5.1414 7594692

[35] Runfola D, Anderson A, Baier H, Crittenden M, Dowker E, Fuhrig S, et al. geoBoundaries: a global database of political administrative boundaries. PLoS One. 2020;15(4):e0231866. doi: 10.1371/journal.pone.0231866 32330167 PMC7182183

[36] Liberia: Constrained individual countries 2020 UN adjusted (100m resolution) from WorldPop. WorldPop. 10.5258/SOTON/WP00683

[37] World Health Organization. Strengthening the assessment of lymphatic filariasis transmission and documenting the achievement of elimination. Geneva: World Health Organization; 2016.

[38] Panda BB, Krishnamoorthy K, Das A, Jain HK, Dixit S, Rahi M, et al. Mini-TAS as a confirmatory mapping tool for remapping areas with uncertain filarial endemicity to exclude/ include for mass drug administration: a report from field validation in India. PLoS One. 2023;18(11):e0293641. doi: 10.1371/journal.pone.0293641 37922274 PMC10624291

[39] Midzi N, Mutsaka-Makuvaza MJ, Phiri I, Palatio K, Bakajika D, Zouré HM, et al. Shrinking the lymphatic filariasis map of Zimbabwe: reassessing the population requiring treatment through confirmatory mapping. Int J Infect Dis. 2025;152:107791. doi: 10.1016/j.ijid.2025.107791 39880359 PMC11873682

[40] Sime H, Gass KM, Mekasha S, Assefa A, Woyessa A, Shafi O, et al. Results of a confirmatory mapping tool for Lymphatic filariasis endemicity classification in areas where transmission was uncertain in Ethiopia. PLoS Negl Trop Dis. 2018;12(3):e0006325. doi: 10.1371/journal.pntd.0006325 29579038 PMC5886699

[41] World bank data bank: population estimates and projections. 2015. https://databank.worldbank.org/source/population-estimates-and-projections

[42] Norwegian Refugee Council/Internal Displacement Monitoring Centre NRC/ IDMC. Global overview 2011: people internally displaced by conflict and violence - Liberia. 2012.

[43] World Health Organization. Lymphatic filariasis: TAS, a manual for national elimination programmes. World Health Organization; 2011.

[44] Jambulingam P, Kuttiatt VS, Krishnamoorthy K, Subramanian S, Srividya A, Raju HKK, et al. An open label, block randomized, community study of the safety and efficacy of co-administered ivermectin, diethylcarbamazine plus albendazole vs. diethylcarbamazine plus albendazole for lymphatic filariasis in India. PLoS Negl Trop Dis. 2021;15(2):e0009069. doi: 10.1371/journal.pntd.0009069 33591979 PMC7909694

[45] Chesnais CB, Awaca-Uvon N-P, Bolay FK, Boussinesq M, Fischer PU, Gankpala L, et al. A multi-center field study of two point-of-care tests for circulating *Wuchereria bancrofti* antigenemia in Africa. PLoS Negl Trop Dis. 2017;11(9):e0005703. doi: 10.1371/journal.pntd.0005703 28892473 PMC5608416

[46] Fimbo AM, Minzi OMS, Mmbando BP, Barry A, Nkayamba AF, Mwamwitwa KW, et al. Prevalence and correlates of lymphatic filariasis infection and its morbidity following mass ivermectin and albendazole administration in Mkinga district, North-Eastern Tanzania. J Clin Med. 2020;9(5):1550. doi: 10.3390/jcm9051550 32455556 PMC7290598

[47] Poindexter HA. Filariasis bancrofti studies in Liberia. Am J Trop Med Hyg. 1950;30(4):519–23. doi: 10.4269/ajtmh.1950.s1-30.519 15425742

[48] World Health Organization. The expanded special project for elimination of neglected tropical diseases (ESPEN). Annual report. 2021. https://espen.afro.who.int/countries/liberia

[49] Kelly-Hope L, Paulo R, Thomas B, Brito M, Unnasch TR, Molyneux D. Loa loa vectors Chrysops spp.: perspectives on research, distribution, bionomics, and implications for elimination of lymphatic filariasis and onchocerciasis. Parasit Vectors. 2017;10(1):172. doi: 10.1186/s13071-017-2103-y 28381279 PMC5382514

[50] World Health Organization. Diagnostic test for surveillance of lymphatic filariasis: target product profile. Geneva: World Health Organization; 2021. https://iris.who.int/bitstream/handle/10665/340081/9789240018648-eng.pdf

[51] Irish SR, Al-Amin HM, Paulin HN, Mahmood ASMS, Khan RK, Muraduzzaman AKM, et al. Molecular xenomonitoring for *Wuchereria bancrofti* in Culex quinquefasciatus in two districts in Bangladesh supports transmission assessment survey findings. PLoS Negl Trop Dis. 2018;12(7):e0006574. doi: 10.1371/journal.pntd.0006574 30048460 PMC6062013

[52] Subramanian S, Jambulingam P, Krishnamoorthy K, Sivagnaname N, Sadanandane C, Vasuki V, et al. Molecular xenomonitoring as a post-MDA surveillance tool for global programme to eliminate lymphatic filariasis: field validation in an evaluation unit in India. PLoS Negl Trop Dis. 2020;14(1):e0007862. doi: 10.1371/journal.pntd.0007862 31978060 PMC7001988

[53] Thomas GS, Liu Y, Mwanga N. Exploring the environmental effects of urbanization in Monrovia. ejtas. 2024;2(3):1117–30. doi: 10.59324/ejtas.2024.2(3).89

[54] Nchoutpouen E, Talipouo A, Djiappi-Tchamen B, Djamouko-Djonkam L, Kopya E, Ngadjeu CS, et al. Culex species diversity, susceptibility to insecticides and role as potential vector of Lymphatic filariasis in the city of Yaoundé, Cameroon. PLoS Negl Trop Dis. 2019;13(4):e0007229. doi: 10.1371/journal.pntd.0007229 30943198 PMC6464241

