# Supplementary material for: Assessing the need to implement mass drug administration against Wuchereria bancrofti infection using both human serology and xenomonitoring in the urban conurbation of Monrovia, Liberia
Source: PLoS Negl Trop Dis. 2025 Sep 18;19(9):e0013446. doi: 10.1371/journal.pntd.0013446 (PMC12445466; doi:10.1371/journal.pntd.0013446)
Supplement: S1 Table — (DOCX) [file pntd.0013446.s001.docx]

| **S1 Table. A) Community catches for Exit Traps** | | | | | | | | | | |  |
| --- | --- | --- | --- | --- | --- | --- | --- | --- | --- | --- | --- |
| **District** | **Community** | **Trap events** | **Total collected** | ***Culex*** | | ***An. gambiae*** | | **Others** | | **Total fed** |  |
|  |  |  |  | **n** | **fed** | **n** | **fed** | **n** | **fed** |  |  |
| Bushrod | Cald well New Georgia | 209 | 1346 | 784 | 79 (10.1%) | 386 | 42 (10.9%) | 176 | 56 (31.8%) | 177 (13.2%) |  |
|  | Clara town | 207 | 786 | 713 | 158 (22.2%) | 47 | 9 (19.1%) | 26 | 10 (38.5%) | 177 (22.5%) |  |
|  | Doe Community (freeport) | 209 | 798 | 715 | 62 (8.7%) | 76 | 9 (11.8%) | 7 | 1 (14.3%) | 72 (9.0%) |  |
|  | Jamaica road | 209 | 692 | 464 | 82 (17.7%) | 202 | 24 (11.9%) | 26 | 1 (3.8%) | 107 (15.5%) |  |
|  | Mombo town | 231 | 1153 | 856 | 214 (25.0%) | 201 | 51 (25.4%) | 96 | 37 (38.5%) | 302 (26.2%) |  |
|  | New Kru town | 201 | 250 | 220 | 97 (44.1%) | 22 | 13 (59.1%) | 8 | 2 (25.0%) | 112 (44.8%) |  |
|  | Point 4 | 272 | 385 | 350 | 83 (23.7%) | 31 | 15 (48.4%) | 4 | 0 (0.0%) | 98 (25.5%) |  |
|  | Tweh farm | 280 | 732 | 645 | 59 (9.1%) | 86 | 16 (18.6%) | 1 | 0 (0.0%) | 75 (10.2%) |  |
| Commonwealth | Casear Town | 185 | 1432 | 1,050 | 219 (20.9%) | 366 | 64 (17.5%) | 16 | 3 (18.8%) | 286 (20.0%) |  |
|  | Cooper's Farm | 162 | 936 | 834 | 191 (22.9%) | 99 | 36 (36.4%) | 3 | 1 (33.3%) | 228 (24.4%) |  |
|  | Cowfield Community | 199 | 492 | 345 | 94 (27.2%) | 141 | 37 (26.2%) | 6 | 4 (66.7%) | 135 (27.4%) |  |
|  | Kpelleh town | 160 | 1326 | 777 | 158 (20.3%) | 528 | 136 (25.8%) | 21 | 1 (4.8%) | 295 (22.3%) |  |
|  | Mount Barclay | 200 | 113 | 67 | 30 (44.8%) | 39 | 28 (71.8%) | 7 | 0 (0.0%) | 58 (51.3%) |  |
|  | Omega | 176 | 1908 | 1,401 | 197 (14.1%) | 481 | 84 (17.5%) | 26 | 5 (19.2%) | 286 (15.0%) |  |
|  | Soul Clinic | 164 | 625 | 526 | 68 (12.9%) | 93 | 13 (14.0%) | 6 | 0 (0.0%) | 81 (13.0%) |  |
|  | Wood camp Community | 162 | 400 | 282 | 76 (27.0%) | 101 | 60 (59.4%) | 17 | 2 (11.8%) | 138 (34.5%) |  |
| **Total** | | **3,226** | **13374** | **10,029** | **1,867 (18.6%)** | **2,899** | **637 (22.0%)** | **446** | **123 (27.6)** | **2,627 (19.6%)** |  |

**S1 Table. B) Community catches for Gravid Traps**

| **District** | **Community** | **Trap events** | **Total collected** | ***Culex*** | | ***An. gambiae*** | | **Others** | | **Total fed** |
| --- | --- | --- | --- | --- | --- | --- | --- | --- | --- | --- |
|  |  |  |  | **n** | **fed** | **n** | **fed** | **n** | **fed** |  |
| Bushrod | Cald well New Georgia | 25 | 207 | 198 | 114 (57.6%) | 7 | 0 (0.0%) | 3 | 2 (66.7%) | 116 (56.0%) |
|  | Clara town | 24 | 442 | 436 | 174 (39.9%) | 4 | 0 (0.0%) | 33 | 2 (6.1%) | 176 (39.8%) |
|  | Doe Community (freeport) | 23 | 519 | 514 | 115 (22.4%) | 5 | 1 (20.0%) | 15 | 0 | 116 (22.4%) |
|  | Jamaica road | 23 | 450 | 441 | 159 (36.1%) | 8 | 2 (25.0%) | 18 | 1 (5.6%) | 162 (36.0%) |
|  | Mombo town | 25 | 412 | 407 | 226 (55.5%) | 5 | 2 (40.0%) | 50 | 0 | 228 (55.3%) |
|  | New Kru town | 24 | 430 | 430 | 225 (52,3%) | 0 | 0 (0.0%) | 2 | 0 | 225 (52.3%) |
|  | Point 4 | 30 | 293 | 293 | 101 (34.5%) | 0 | 0 (0.0%) | 6 | 0 | 101 (34.5%) |
|  | Tweh farm | 30 | 201 | 201 | 106 (52.7%) | 0 | 0 (0.0%) | 1 | 0 | 106 (52.7%) |
| Commonwealth | Casear Town | 26 | 217 | 214 | 140 (65.4%) | 3 | 1 (33.3%) | 9 | 0 | 141 (65.0%) |
|  | Cooper's Farm | 23 | 1,705 | 1,694 | 123 (7.3%) | 11 | 0 (0.0%) | 2 | 0 | 123 (7.2%) |
|  | Cowfield Community | 28 | 235 | 235 | 143 (60.9%) | 0 | 0 (0.0%) | 10 | 0 | 143 (60.9%) |
|  | Kpelleh town | 23 | 272 | 270 | 207 (76.7%) | 2 | 0 (0.0%) | 19 | 0 | 207 (76.1%) |
|  | Mount Barclay | 28 | 237 | 234 | 132 (56.4%) | 3 | 2 (66.7%) | 37 | 0 | 134 (56.5%) |
|  | Omega | 25 | 345 | 280 | 85 (30.4%) | 65 | 3 (4.6%) | 4 | 0 | 88 (25.5%) |
|  | Soul Clinic | 24 | 210 | 207 | 123 (59.4%) | 3 | 0 (0.0%) | 8 | 0 | 123 (58.6%) |
|  | Wood camp Community | 24 | 258 | 252 | 225 (89.3%) | 5 | 2 (40.0%) | 69 | 1 (1.4%) | 228 (88.4%) |
| **Total** |  | **405** | **6,433** | **6,306** | **2,398 (38.0%)** | **121** | **13 (10.7%)** | **286** | **6 (2.1%)** | **2,417 (37.6%)** |
